# Novel base-pairing interactions at the tRNA wobble position crucial for accurate reading of the genetic code

**DOI:** 10.1038/ncomms10457

**Published:** 2016-01-21

**Authors:** Alexey Rozov, Natalia Demeshkina, Iskander Khusainov, Eric Westhof, Marat Yusupov, Gulnara Yusupova

**Affiliations:** 1 Department of Integrated Structural Biology, Institute of Genetics and Molecular and Cellular Biology, INSERM, U964; CNRS/University of Strasbourg, UMR7104, 1 rue Laurent Fries, BP 10142, Illkirch 67404, France; 2 Institute of Fundamental Medicine and Biology, Kazan Federal University, Karl Marx 18, Kazan 420012, Russia; 3 Architecture and Reactivity of RNA, Institute of Molecular and Cellular Biology of the CNRS, University of Strasbourg, UPR9002, 15 rue Rene Descartes, Strasbourg 67084, France

## Abstract

Posttranscriptional modifications at the wobble position of transfer RNAs play a substantial role in deciphering the degenerate genetic code on the ribosome. The number and variety of modifications suggest different mechanisms of action during messenger RNA decoding, of which only a few were described so far. Here, on the basis of several 70S ribosome complex X-ray structures, we demonstrate how *Escherichia coli* tRNA^Lys^_UUU_ with hypermodified 5-methylaminomethyl-2-thiouridine (mnm^5^s^2^U) at the wobble position discriminates between cognate codons AAA and AAG, and near-cognate stop codon UAA or isoleucine codon AUA, with which it forms pyrimidine–pyrimidine mismatches. We show that mnm^5^s^2^U forms an unusual pair with guanosine at the wobble position that expands general knowledge on the degeneracy of the genetic code and specifies a powerful role of tRNA modifications in translation. Our models consolidate the translational fidelity mechanism proposed previously where the steric complementarity and shape acceptance dominate the decoding mechanism.

## Abstract (web summary)

The anticodon loops of almost all tRNAs contain modifications known to be important for their function. Here the authors use crystallography to provide new mechanistic insights into how the modification at the wobble position of the *E. coli* tRNALys_UUU_ assists in discrimination between cognate and near-cognate codons.

## Graphical abstract

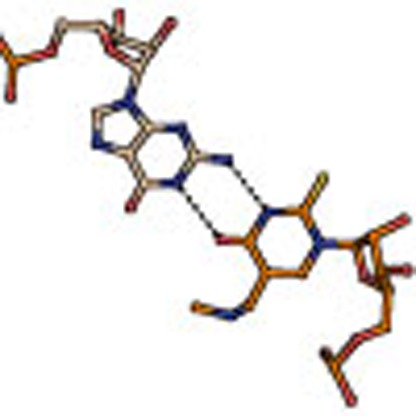

More than a hundred of posttranscriptional RNA modifications identified today^1^ were shown to play diverse and indispensable roles in gene regulation in all domains of life^2^. Modifications of RNA are carried out by complex cellular pathways, which involve countless protein enzymes and catalytic RNA–protein complexes, which primarily target tRNAs and, to a lesser extent, ribosomal RNA and mRNAs^1^. The observed trends suggest that many modification motifs and their sequence locations are conserved throughout Bacteria, Archaea and Eukarya; however, some kingdom-specific differences are documented as well.

Among all modifications found, those of tRNA are the most abundant and studied classes of modifications^1^,^3^,^4^,^5^. Ninety-three tRNA modifications described today represent an astonishing library of chemically diverse structures^5^ each of which influences in a unique way three-dimensional integrity of the tRNA and specifies its physicochemical properties^6^,^7^. The most elaborate tRNA modifications are located in the anticodon loop, which comprises the anticodon triplet necessary for pairing with mRNA codons. The anticodon loops of almost all tRNAs contain several modified nucleotides. Among these, the most important are nucleotides in the positions 34 and 37. The nucleotide in position 34 (so-called ‘wobble' position) pairs with the third mRNA codon base in the aminoacyl-tRNA binding site (A-site) during decoding^4^,^8^. The nucleotide in the position 37 is adjacent to the 3′-side of the anticodon.

From the moment of their discoveries, modifications in tRNA anticodon loops were demonstrated to be crucial for proper mRNA decoding and fine-tuning of the process^9^. In particular, modifications of tRNA position 34 were implied to increase tRNA capacities to decode multiple mRNA codons differing by the third nucleoside (synonymous codons), hence explaining how the degenerate genetic code is translated^4^,^10^,^11^. It was shown also that anticodon modifications enhance recognition by corresponding aminoacyl-tRNA synthetases^12^,^13^ and serve as a preventing measure of frame shifting during translocation^14^,^15^. Very recently, the tRNA modifications were also credited a role in connecting translation, metabolism and stress response in bacteria and eukaryotes^2^,^16^. For example, in humans the deregulation of RNA modification pathways was shown to be linked to the type two diabetes and several mitochondrial diseases^16^.

Except for methionine and tryptophan, all amino acids are encoded by more than one codon, because the genetic code is redundant^17^,^18^ with 61 codons encoding 20 amino acids. In contrast to eukaryotes, where synonymous codons AAA and AAG are read by three isoacceptor lysine tRNA^Lys^, half of all bacteria have only one isoacceptor tRNA^Lys^_UUU_ that decodes these two codons into amphipathic amino acid lysine^9^,^19^,^20^. To discriminate against pyrimidine-ending codons AAC and AAU encoding asparagine, the *E. coli* tRNA^Lys^_UUU_ contains one of the most complex modifications 5-methylaminomethyl-2-thiouridine (S, mnm^5^s^2^U) at the wobble anticodon position 34 (Fig. 1a). The mnm^5^s^2^ modification is a result of a sophisticated pathway that includes many enzymes responsible for thiolation and attachment of a methylaminomethyl group^21^. Another prominent feature of *E. coli* tRNA^Lys^_SUU_ is N6-threonylcarbamoyladenosine (t^6^A) at the 37th position of its anticodon loop (Fig. 1a). The t^6^A modification is one of the most ubiquitous and conserved, and is known to be critical for recognition of codons starting with adenosine. This is one of the rare modifications universally conserved throughout different kingdoms of life^1^. In addition to S34 and t^6^A37, *E. coli* tRNA^Lys^_SUU_ anticodon loop bears the third modification, pseudouridine at position 39 (Fig. 1a).

Nuclear magnetic resonance studies of unmodified, partially and fully modified anticodon stem loops (ASLs) of *E.coli* tRNA^Lys^_SUU_ demonstrated that mnm^5^s^2^U and t^6^A modifications remodel an otherwise dynamic loop to canonical open U-turn structure to perfectly adapt in the ribosomal decoding centre^6^,^22^. First, X-ray studies of the partial decoding system^23^, where crystals of the isolated small ribosomal 30S subunit were soaked with synthetic ASL of *E.coli* tRNA^Lys^_UUU_ and hexaribonucleotides as mRNA analogues, shed some light on possible roles of modifications in decoding^24^,^25^. It was suggested that t^6^A37 enhances the codon–anticodon stability via cross-strand stacking interaction with the first codon nucleotide, whereas partially modified mnm^5^U34 lacking 2-thio group was implicated in an alternate mnm^5^U34·G base-pairing interactions via a bifurcated hydrogen bond^25^. A similar model of the bacterial 30S subunit with ASL of human tRNA_3_ ^Lys^_UUU_, which has identical anticodon loop sequence with *E. coli* tRNA^Lys^_SUU_ but carries ms^2^t^6^A37 (2-methylthio-N6-threonylcarbamoyladenosine) and mcm^5^s^2^U34 (5-methoxycarbonylmethyl-2-thiouridine) modifications, revealed Watson–Crick-like geometry of mcm^5^s^2^U34·G base-pairing interactions at the wobble codon–anticodon position^26^. Both of these works and numerous other genetic, biophysical and biochemical findings indicated that each group in a modified nucleotide improves thermodynamic properties of tRNA and serves to augment specific codon–anticodon interactions during decoding.

Recent crystallographic studies of the 30S ribosomal subunit or complete 70S ribosome complexes with different ASLs^23^,^24^,^25^,^26^,^27^ or tRNAs^28^,^29^,^30^,^31^,^32^,^33^ and mRNAs describe three major classes of preferred geometry at the wobble position of a codon–anticodon minihelix. The first class consists of canonical Watson–Crick purine–pyrimidine A·U (or U·A) and C·G (or G·C) pairs^29^,^30^,^33^, and also includes Watson–Crick-like pairs. It was suggested that the latter closely resemble canonical geometry via stabilization of an enol tautomer by the wobble modifications (for example, above described mcm^5^s^2^U34·G)^26^,^27^. The second class consists of standard wobble pair G34·U originally predicted by Crick^8^. In this pair, the pyrimidine is displaced towards the major groove of the codon–anticodon minihelix; however, the distance between the ribose C1′ atoms remains very close to 10.5 Å, the average value for a standard Watson–Crick pair^28^,^29^. The third class includes I·G or I·A pairs, where I (inosine) is present at position 34 of some tRNAs^1^,^34^. These purine–purine pairs are unusually wide with a C1′–C1′ distance of 12.3 Å (refs 24, 32).

In the current study, we describe six X-ray structures of physiologically relevant complexes of the complete 70S ribosome primed with long mRNAs that contain full-length native *E. coli* tRNA^fMet^ in the peptidyl-tRNA-binding site (P-site) and tRNA^Lys^_SUU_ bound to cognate or near-cognate codons in the A-site. We identified an unprecedented base-pairing interaction at the wobble position of the codon–anticodon duplex in the decoding centre that broadens the present family of ‘wobble geometries'. This base pair, which involves a hypermodified S34 of *E. coli* tRNA^Lys^_SUU_ and codon guanosine, represents a ‘wobble' (G34·U) with the U moved towards the minor instead of the major groove that is much less isosteric to its flipped form than usual wobble G·U pair.

The ribosome structures we are describing in this work deepen the understanding of the tRNA discrimination mechanism on the ribosome. We demonstrate how tRNA^Lys^_SUU_ discriminates between cognate codons AAA and AAG, and the near-cognate stop codon UAA (ochre codon) or the isoleucine codon AUA, with which it forms pyrimidine–pyrimidine U·U mismatches. Together with our earlier structures of the 70S ribosome with various mismatches in the codon–anticodon duplex^29^,^30^, the present models expand our library of various states of the 70S decoding centre. The present evidence further strengthens our proposition that the steric complementarity is predominant over the number of hydrogen bonds^35^ between the decoding centre and the codon–anticodon duplex, and hence plays the crucial discriminatory role during decoding.

## Results

### The modifications of lysine tRNA in the 70S decoding centre

**Figure 1:**
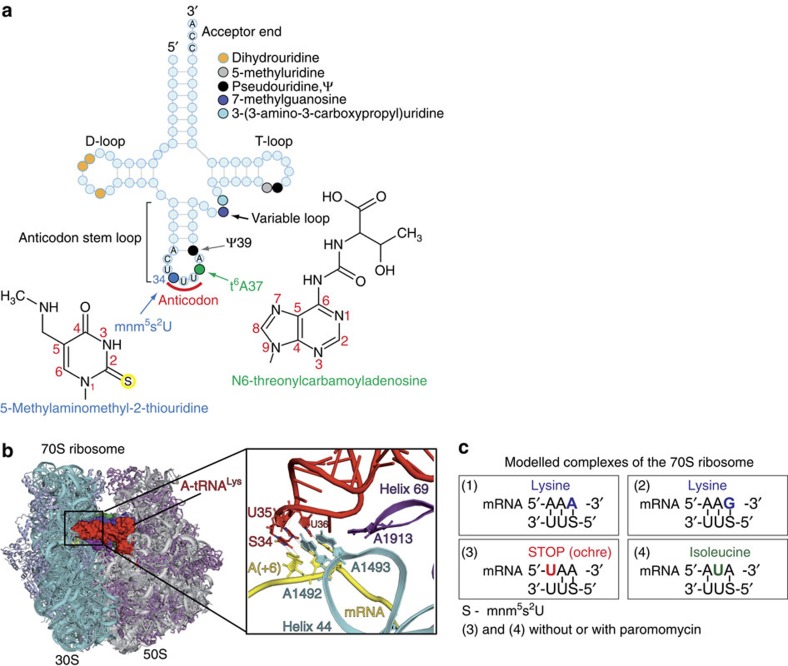
Structure and modifications of lysine tRNA^Lys^_SUU_ from *E. coli* and complexes of the 70S ribosome with tRNA^Lys^_SUU_ in the A-site. (**a**) Secondary structure of *E. coli* tRNA^Lys^_SUU_. Major domains and modifications of tRNA are indicated; chemical formulas of hypermodified nucleobases at positions 34 and 37 are given together with corresponding abbreviations. (**b**) Side view of the 70S ribosome complex with three tRNAs bound at the A- (red), P- (blue) and exit (green) binding sites. Helix 69 of the large subunit is in magenta. The frame designates the decoding centre with the bound anticodon-stem loop of tRNA^Lys^_SUU_ and the close-up view on the codon–anticodon duplex and major nucleotides of the decoding centre (G530 from 16S rRNA is not shown) including A1913 from 23S rRNA. (**c**) Schemes of codon–anticodon duplexes in the decoding centre of the 70S ribosome complexes modelled in the study. The complexes are numbered in accordance with description in the main text.

In this work as in our previous studies^28^,^29^,^30^, we employed the full *Thermus thermophilus* 70S ribosome co-crystallized with long synthetic mRNAs and natural tRNAs. These complexes model cognate or near-cognate states of the decoding centre at the proofreading step of the tRNA selection process (Fig. 1b). We determined six X-ray structures of the 70S ribosome programmed by 30-nucleotide-long mRNAs with AUG codon and *E. coli* tRNA^fMet^ in the P-site and the A-site occupied by tRNA^Lys^_SUU_ bound to its cognate codon AAA or AAG, or near-cognate stop codon UAA and isoleucine codon AUA (Fig. 1c and Table 1).

**Figure 2:**
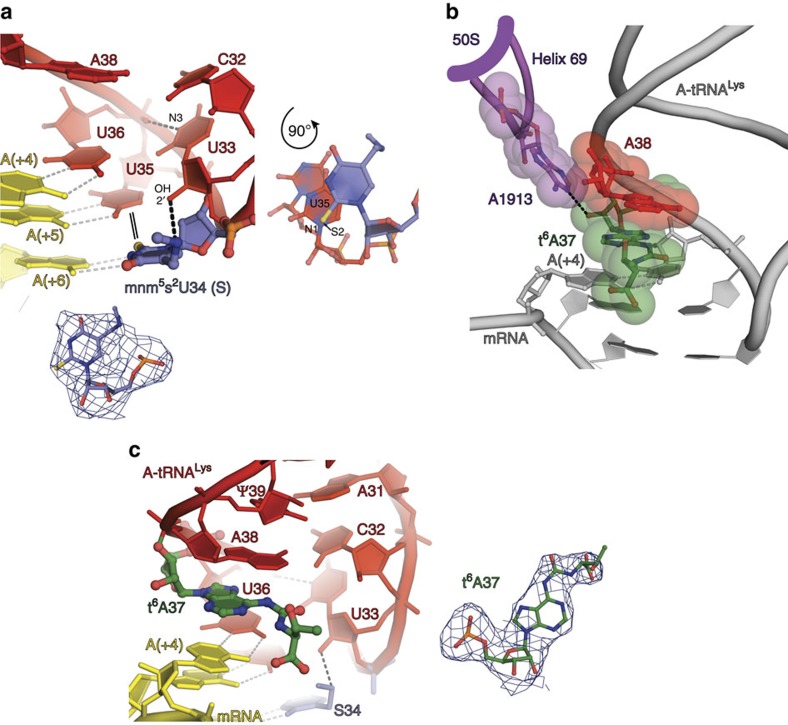
Details of *E. coli* tRNA^Lys^_SUU_ anticodon loop interactions. (**a**) The S34 interactions: stabilization of the U-turn structure via anchoring of the mnm^5^ group and stacking of the thio group with the N1 atom of U35 shown in an alternative top-view orientation on the right. Nucleotide 37 is omitted for clarity of representation. (**b**) The invariant A1913 in Helix 69 of 23S rRNA of the large ribosomal subunit (thick magenta line) constrains position of the t^6^A37 ribose by conserved hydrogen bond interactions; van der Waals surfaces show that A1913 also defines conformation of the anticodon loop from the 3′-side of t^6^A37 at position 38. (**c**) Cross-strand stacking of t^6^A37 with the first nucleotide of the A-site bound codon (position (+4)). In **a** and **c**, *2F*_o_−*F*_c_ electron density maps corresponding to S34 and t^6^A37 are contoured at 1.0*σ*.

The electron density maps of the two cognate complexes (Fig. 1c, complexes 1 and 2) possess sufficient level of detail to discern structural features of the tRNA anticodon loops that can be attributed to the influence of modifications mnm^5^s^2^U and t^6^A (Fig. 2). The amino group of the modified nucleotide S34 forms a hydrogen bond with 2′-OH of nucleotide U33 (Fig. 2a), thus altering the U-turn structure and stabilizing it, as was shown before for isolated ASLs^6^. The thio group of the same nucleotide is known to stabilize 3′-endo conformation of the ribose favourable for base-pairing interactions^36^, influencing the codon–anticodon helix stability. We observed a feature, characteristic of 2-thiouridine^37^ as well, namely the S2(S34)-N1(U35) ‘stacking' interaction with the subsequent nucleotide U35 (Fig. 2a). This interaction affects the relative positioning of the S34 and U35 nucleotides, and hence the shape and stability of the codon–anticodon duplex.

An influence of the large 50S ribosomal subunit on the tRNA constraints during decoding remained underestimated for a long time, because the first models of decoding were based on the structures of the isolated small ribosomal subunit^23^,^35^. On the 70S ribosome, the conserved helix 69 of the 50S subunit, which is pivotal for many functions of the ribosome, directly contacts the sugar moiety of the tRNA nucleotide at position 37 (refs 31, 38 and Fig. 2b). Most probably, this contact is important for proper positioning and conformational stabilization of the anticodon loop. In addition, t^6^A37 forms cross-strand stacking with the first nucleotide of the mRNA codon in the A-site (Fig. 2c). Similar stacking interactions were described in the early models of the 30S subunit whose crystals were soaked with the tRNA^Lys^_UUU_ ASL carrying t^6^A37 and mnm^5^U34 modifications^25^. However, the comparison of our present structure with this model revealed a considerable shift of 1 Å in the position of the t^6^A37 nucleotide pointing to a specific role of the large 50S subunit in restraining the anticodon loop of the A-site bound tRNA (Supplementary Fig. 1). The position of t^6^A37 over the A·U pair itself (Fig. 2c), as observed in our structures, corresponds well with the main function of this modification in stabilizing the weak A·U base-pairing interactions and preventing mRNA slippage during translocation as well.

### Hypermodified uridine forms a unique base pair

The capacity of *E. coli* tRNA^Lys^_SUU_ to read both codons AAA and AAG ending with purines implies that the hypermodified uridine S34 at the first anticodon position is involved in a dual mode of base-pairing interactions with adenosine and guanosine. In general, in bacteria the AAA codon is used approximately three times more often than the AAG codon^39^. However, it was estimated that when the next codon after the one encoding lysine starts with cytidine the AAG codon becomes preferred^40^.

**Figure 3:**
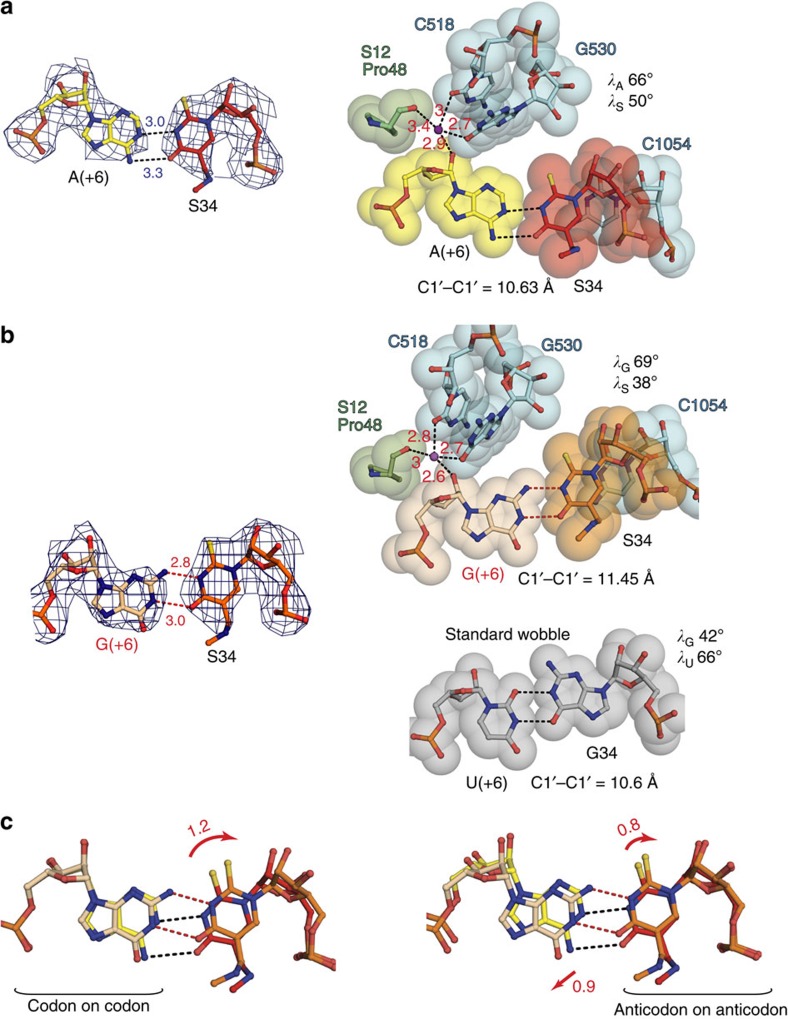
Base pairs at the wobble position of the codon–anticodon duplex formed by the SUU anticodon of *E. coli* tRNA^Lys^_SUU_ on cognate codons AAA or AAG. (**a**) The S34·A(+6) pair. Left, hydrogen bonds are indicated (Å); right, van der Waals surfaces with the corresponding C1′–C1′ distance and glycosidic angles (*λ*). (**b**) The S34·G(+6) pair; indicated parameters as in **a**; possible hydrogen bonds are marked by red dashes. In **a** and **b**, the sugar moiety of codon nucleosides is coordinated by magnesium ion (shown in magenta) together with small subunit elements (Pro 48 of S12 and C518 with G530 in 16S rRNA); coordination distances show that the position of the wobble nucleotide in the codon can slightly adjust depending on the type of pairing interactions. The anticodon nucleotide is weakly restricted by C1054 in 16S rRNA via lone pair–aromatic interactions. The lower panel displays the standard wobble G34·U(+6) pair with specified parameters as in **a** and **b**; see PDB code: 3H8I. (**c**) Superposition of S34·A(+6) and S34·G(+6) pairs by the mRNA codons (left) or by the tRNA^Lys^_SUU_ anticodon (right) shows relative extent of the guanosine and modified uracil displacement in the S34·G(+6) pair; the displacement is estimated by angstroms and marked by red arrows. In **a** and **b** *2F*_o_−*F*_c_ electron density maps are contoured at 1.5*σ*.

Our first structure with *E. coli* tRNA^Lys^_SUU_ bound to its cognate AAA codon showed that hypermodified uridine S34 formed a distorted Watson–Crick base pair with the opposing adenosine with the standard C1′–C1′ distance of 10.6 Å (Fig. 3a). The slight positional deviation of the uracil ring from its standard position in the Watson–Crick A·U pair, caused by the interactions of the modifications with neighbouring nucleotides U33 and U35 (Fig. 2a), resulted in weakening of interaction between the codon adenosine and S34. On the other hand, the very same interactions tend to strengthen the codon–anticodon duplex as a whole by adjustment of the shape of tRNA U-turn^6^ (Fig. 2a).

The model of tRNA^Lys^_SUU_ bound to its second cognate codon AAG demonstrated an unprecedented and striking base-pairing geometry (Fig. 3b). The new S34·G(+6) pair is characterized by the larger C1′–C1′ distance of 11.5 Å that exceeds a corresponding distance in a standard Watson–Crick pair by 1 Å. Yet, interatomic distances between Watson–Crick edges within S34·G(+6) imply the existence of two hydrogen bonds between carbonyl oxygen and N3 atom of modified uracil, and N3 atom and amino group of guanosine, respectively (Fig. 3b).

**Figure 4:**
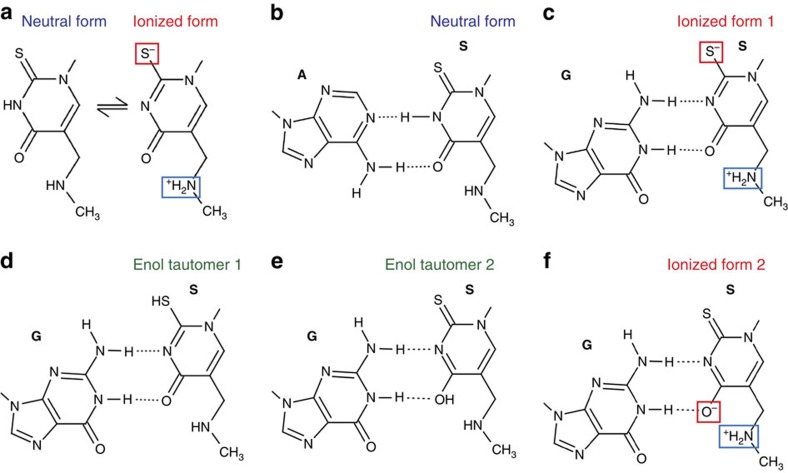
The chemical basis of observed base-pairing interactions of S34 with the third codon adenosine or guanosine. (**a**) Equilibrium between two forms of mnm^5^s^2^U^41^. (**b**) Interactions with adenosine of the neutral form of mnm^5^s^2^U. (**c**) Theoretically predicted base pair with guanosine of zwitterionic form of mnm^5^s^2^U carrying negative charge on sulfur atom. (**d**,**e**) Two alternative pairing interactions with guanosine and enol tautomeric forms of mnm^5^s^2^U. (**f**) Base-pairing interactions with guanosine of a possible zwitterionic form of mnm^5^s^2^U.

One of the hypotheses that could explain observed pairing interactions suggested that under physiological conditions a significant fraction of mnm^5^s^2^U is present in a zwitterionic form (Fig. 4a)^41^. This is due to the increased acidity of the N3 proton by the inductive effect of the protonated methylaminomethyl group, as was predicted based on the theoretical estimate of the p*K*_a_ (ref. 41). In its neutral form, the modified uracil forms two hydrogen bonds same as in the case for a standard Watson–Crick U·A pair and as we observed for the mnm^5^s^2^U34·A(+6) pair (Fig. 4b). In its deprotonated form, the N3 atom of the uracil becomes a proton acceptor and can form a hydrogen bond with the amino group of guanosine, while the uracil carboxyl group is engaged in another hydrogen bond with the guanosine N3 atom (Fig. 4c). Although less probable, the observed unusual pattern of hydrogen bonds between the modified uridine and guanosine can also be rationalized by existence of either rare tautomeric states (Fig. 4d,e) or an alternative zwitterionic state (Fig. 4f) of the modified uridine.

It is worth to underline here that in contrast to the first two codon–anticodon positions, which are tightly restricted by the decoding centre^29^,^30^, the ‘wobble' pair is not very firmly stabilized. The codon nucleotide is held in place only by indirect interactions with G530, C518 and S12 through a Mg^2+^ ion (Fig. 3a,b) and the O4' of the first anticodon nucleotide forms weak off-centre lone pair–π interaction with the nucleobase of C1054 in 16S rRNA (Supplementary Fig. 2)^42^. However, the fact that observed S34·G(+6) pair is distorted from the standard ‘wobble' geometry suggests that the aforementioned indirect restraints and interactions of the modification groups provide some restraints to control geometry of the third base pair.

### Pyrimidine–pyrimidine mismatch in the 70S decoding centre

The translation of genes into proteins is an error-prone process with the average frequencies of mistranslation 10^−3^–10^−5^ (ref. 43). We have recently published first structural rationales for the phenomenon of translational infidelity^30^. We demonstrated that because the G·U mismatches can mimic the form of a canonical Watson–Crick pair via tautomerization or ionization, these type of mismatches become accepted in the decoding centre, which restricts geometries of allowed pairs to canonical interactions. At the same time, our models with the A·A and C·A mismatches at the first two positions of the codon–anticodon duplex suggested that these pairs would be efficiently discriminated against because of (i) steric clashes within a mispair or of a mispair with the tight decoding centre itself, or (ii) because of absence of stable pairing interactions between pairing nucleotides^29^,^30^.

**Figure 5:**
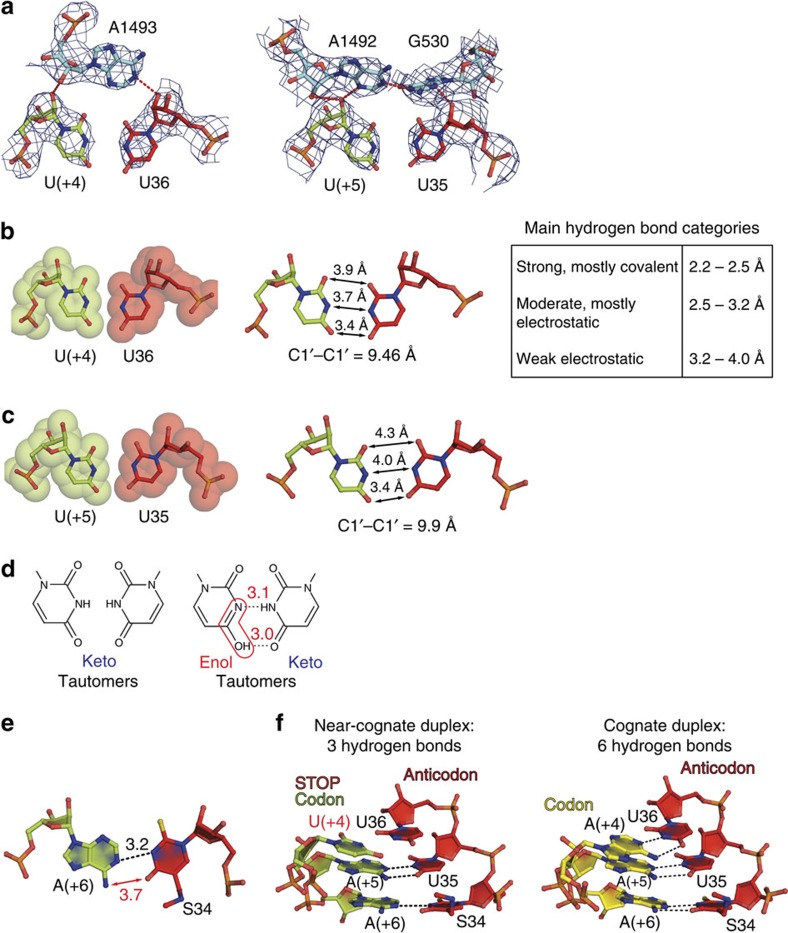
Discrimination basis against near-cognate duplexes with pyrimidine–pyrimidine U·U mismatches in the decoding centre of the 70S ribosome. (**a**) Critical A1492 and A1492/G530 of 16S rRNA stabilize the sugar phosphate backbones of the U·U mismatch at the first (left) and second (right) positions of the codon–anticodon duplex by A-minor groove interactions as is the case for any canonical Watson–Crick pair^29^,^30^. 2*F*_o_−*F*_c_ electron density maps are contoured at 1.5*σ*. (**b**,**c**) Geometries of the U·U mismatch at the first (**b**) and second (**c**) positions of the codon anticodon duplex; van der Waals surfaces (left) together with interatomic distances (right) are presented. In **b**, the table describes standard categories of hydrogen bonds. (**d**) Formation of a strong U·U pair would necessitate a shift in the keto-enol equilibrium from abundant keto form (left panel) to a rare enol form (red frame) and the 3-Å distance between Watson–Crick surfaces of opposing uridines (right panel). (**e**) Conformation of the wobble S34·A(+6) pair in the complex 3 (see Fig. 1c). The absence of two hydrogen bonds expected for the mnm^5^s^2^U·A pair can reflect (i) a deformation of the codon–anticodon minihelix induced by the mismatch and (ii) the specific influence of the tRNA modification at position 34 known to counteract misreading of the genetic code. (**f**) The near-cognate duplex composed of the tRNA^Lys^_SUU_ anticodon and the ochre stop codon is significantly weakened compared with the cognate version on the AAA codon with the full set of canonical Watson–Crick interactions. The described weakening of the near-cognate duplex would imply dissociation of tRNA^Lys^_SUU_ from the ribosome or, in other terms, rejection.

In the current study, we asked an ensuing question of what is the structural basis for discrimination against the pyrimidine–pyrimidine U·U pair, which represents a low-probability mistake during translation^44^,^45^,^46^. Thus, we determined structures of complexes 3 and 4 (Fig. 1c) where the SUU anticodon of tRNA^Lys^_SUU_ formed a U·U mismatch either with the first or the second codon position. As was anticipated, the nucleotides, critical for decoding A1493 and A1492/G530 of 16S rRNA^47^, stabilized the U·U mismatch at the first and the second codon–anticodon positions via A-minor groove interactions (Fig. 5a). These results substantiated our expanded mechanism of decoding that provides structural basis for discrimination in favour of correct tRNAs and against incorrect tRNAs, and describes identical rearrangements of the decoding centre on binding of cognate or near-cognate tRNA^29^,^30^. In both complexes, the interatomic distances between the Watson–Crick edges of opposing uracils exceeded 3.4 Å, implying weak electrostatic interactions (Fig. 5b,c). A hypothetical U·U pair possible through a shift in the keto-enol equilibrium would require a typical distance of 3.0–3.1 Å between uracils for hydrogen bonds to occur (Fig. 5d). However, positions of uracils in both models make this scenario unlikely (Fig. 5b,c). To put it simply, the restraints on the sugar-phosphate backbones of the codon–anticodon helix imposed by the decoding centre are strong enough to prevent the uracils to come close enough to interact strongly via hydrogen bonds (Supplementary Fig. 3).

In complex 3 with the stop codon UAA and the first U·U mismatch, the codon–anticodon duplex was additionally weakened at the 3′-end where the S34·A(+6) pair was slightly deformed (Fig. 5e). As a result, only three strong hydrogen bonds were formed between codon and the anticodon compared with the cognate version with six hydrogen bonds (Fig. 5f). These results demonstrated why tRNA^Lys^_SUU_ normally does not read the ochre codon. Two additional structures of the near-cognate complexes 3 and 4 (Fig. 1c) solved in the presence of antibiotic paromomycin, which stimulates miscoding, supported our previous conclusion of the antibiotic mechanism of action^48^. In both cases, paromomycin stabilized A1492 and A1493 in the ‘out' from the interior of helix 44 positions, hence stimulating A-minor groove interactions with the first two nucleotides of the A-codon. In addition, binding of the antibiotic led to a positional shift of the A1493 phosphate, resulting in partial alleviation of the restrictive decoding centre from the side of the mRNA codon. Finally, in the ribosome structures with paromomycin we observed a displacement of helix 69 of the large subunit towards the D-stem of tRNA^Lys^_SUU_ that, most probably, enhanced stabilization of a near-cognate substrate on the ribosome.

It is important to mention that uracils in a mismatch at the first codon–anticodon position were closer to each other than at the second position (Fig. 5b,c). In the light of kinetic description of the tRNA selection process on the ribosome, it can be interpreted as the second codon–anticodon position being more controlled than the first one^43^. Accordingly, kinetic evaluations of codon readings by tRNA^Lys^_UUU_ in bacteria assigned the highest accuracy values for the second position in the codon–anticodon duplex^45^. Thus, it is important to indicate here that despite of the fact that we could crystallize described near-cognate states of the ribosome at the proofreading step, in solution these complexes would be prone for dissociation because of a prominent lack of pairing codon–anticodon interactions.

## Discussion

Fifty years ago, Francis Crick suggested some rules for translation of the genetic code on the ribosome and postulated the wobble hypothesis that gave first explanations to degeneracy of the code^8^,^18^. It was predicted that the first two positions of the codon would pair with the anticodon using the standard base pairs, while in the base pairing of the third codon base ‘there is a certain amount of play, or wobble, such that more than one position of pairing is possible'. First examples of foretold wobble non-standard pairs included G·U, U·G, I·A, I·C and I·U pairs, where the nucleoside on the left designates position 34 in tRNA^8^. Since those times, a titanic work on deciphering the genetic code resulted into a simple textbook chart where most of the codons are two- or fourfold degenerate, meaning that one amino acid can be coded by two or four codons differing by the third base. Further identification of tRNA modifications and especially those at the first anticodon ‘wobble' position 34 elaborated more on the phenomenon of degeneracy. The ‘modified wobble hypothesis' suggested that specific tRNA base modifications evolved to discriminate particular codons—expanding and facilitating an ability of tRNA to read more than one codon in some cases and preventing misreading in other cases^4^.

In the present study, we describe a new type of a base pair at the third wobble position of a codon–anticodon duplex in the 70S ribosome decoding centre. Our structures demonstrate that the reversed ‘wobble' pair S34·G(+6) adopts its own geometry, different from the standard G34·U(+6) pair^8^ at the third codon–anticodon position (Fig. 3b). This is possibly due to both certain restraints put on the third base pair by the decoding centre of the 70S ribosome and modifications on the nucleotide S34 of tRNA^Lys^_SUU_, shaping the codon–anticodon helix and the ASL of the tRNA (see Results). In spite of the fact that S34·G(+6) is not isosteric to the standard wobble pair, there is a certain similarity of the overall shape between these two pairs.

Both pairs are characterized by displacement of the anticodon nucleotide—S34 in the S34·G(+6) pair and G in the G34·U(+6) pair—towards the minor groove of the codon–anticodon minihelix. This is achievable because of the apical location of the nucleotide in the U-turn structure of the tRNA anticodon loop^49^. On the codon side of the pairs there is another tendency of shifting; however, in this case it is towards the major groove of the minihelix. Results reported in this study particularly show this displacement (Fig. 3c right). Hence, a term ‘wobble' can be also applied to the capacity of the third codon nucleotide to adjust its position because of difference in strength and type of restraints imposed by the decoding centre on the third base pair as compared with the first and second base pairs (Fig. 3a,b).

Uridine at the wobble positions of various tRNAs is almost always modified in bacteria and eukaryotes^1^,^5^. In many cases, tRNAs with the modified uridines read two codons ending with purine A or G and, in some rare cases, modifications help to recognize all four nucleotides A, G, C and U at the third codon position^50^. Previously, it was shown that preferential form of the third wobble pair with a fully modified uridine approached a standard Watson–Crick-like geometry if this uridine was paired with guanosine^26^,^27^ (Supplementary Fig. 4). Thus, in the partial and heterologous model of the isolated bacterial 30S ribosomal subunit, whose crystals were soaked with an ASL of human tRNA_3_ ^Lys^_UUU_, the modified mcm^5^s^2^U34 formed C·G-like mcm^5^s^2^ U34·G pair^26^. In this pair, the uridine base was shifted towards the major groove when compared with geometry of the mnm^5^s^2^U·G(+6) pair in our model (Supplementary Fig. 4b left). In another study, uridine-5-oxyacetic acid at the position 34 of tRNA^Val^ displayed a similar Watson–Crick-like pair^27^ with guanosine greatly displaced towards the minor groove of a codon–anticodon helix when compared with our structure (Supplementary Fig. 4c left). On the basis of these 30S models, it was reasoned that modifications stabilized enol tautomers of uracil to keep the Watson–Crick-like geometry. It should also be mentioned here that although these structures gave important insights into how tRNA modifications can influence base-pairing interactions in the decoding centre, they described a partial system of decoding where roles of a full-length tRNA and the large ribosomal subunit could not be taken into consideration. At the same time, proper understanding of most often subtle fine-tuning effects of tRNA modifications on the codon–anticodon pairing geometries would require advanced experimental system consisting of full-length ligands and complete ribosome.

The novel pairing interaction at the third position of the codon–anticodon duplex described in this work deepens our understanding of principles embedded into translation of the genetic code on the ribosome. In agreement with the ‘modified wobble hypothesis', our data show that the shape of the ‘wobble' base pair is jointly defined by the ribosome environment and the tRNA modifications. In the observed case, such synergy gave rise to the novel base-pairing pattern, never observed before in the tRNA–mRNA duplexes. From the observation we can derive both a wider spatial tolerance of the wobble base-pair environment than expected before and, on the other hand, a certain degree of strictness imposed on the base pair, forcing it into the conformation unusual for a relaxed duplex.

Together with our preceding models describing G·U, U·G, A·C and A·A mismatches in codon–anticodon duplexes bound in the 70S decoding centre, the present models with pyrimidine–pyrimidine U·U mismatches consolidate the translation fidelity mechanism put forward by us earlier^29^,^30^. In this mechanism, the ribosome responds identically on binding of cognate or near-cognate tRNA by enveloping the codon–anticodon duplex in a rigid universal mould of the ‘closed' decoding centre, which favours the Watson–Crick geometry of codon–anticodon base pairs. It is crucial to emphasize that when near-cognate tRNA with a mismatch to the mRNA codon binds to the decoding centre (during initial selection and proofreading steps), the number of hydrogen bonds between the minor groove of the near-cognate codon–anticodon helix and the ‘closed' decoding centre elements is the same as would be the case during binding of the cognate tRNA. Therefore, the ribosome is incapable to distinguish between these states by the minor groove geometry of codon–anticodon base pairs. However, in contrast to cognate tRNAs that will stably pair to the mRNA codon by canonical Watson–Crick interactions, near-cognate tRNAs with a mismatch to the codon will be more likely to dissociate from the ribosome because of the strict restraints imposed by the tertiary structure of tRNA and elements of the ‘closed' decoding centre on the codon–anticodon helix. This pressure constitutes the discriminatory force by preventing the following conformational changes: (a) widening of the codon–anticodon helix needed to accommodate bulky non-canonical pairs (for example, A·A pair); (b) narrowing of the codon–anticodon helix necessary for proper pairing interaction in pyrimidine–pyrimidine pairs (for example, U·U pair); or (c) shift of nucleobases towards minor or major groove, characteristic of, for example, wobble G·U pair. The third base pair of the codon–anticodon duplex contributes in a different manner, compared with the first two positions. Its role in decoding is linked to the base pair nature, the indirect restraints imposed by the decoding centre and the presence of the modification groups that influence conformations of the tRNA ASL and the codon–anticodon helix. An additional discriminatory role at the proofreading step can be performed by the tails of some ribosomal proteins that selectively stabilize cognate tRNA substrates^51^. The rare translational mistakes caused by the incorporation of near-cognate tRNAs are reasoned mostly by the ability of some tRNAs to form Watson–Crick-like base pairs via ionization or tautomerism^29^, or in some cases a mismatch randomly escapes discrimination by preserving geometry close to the Watson–Crick pair^30^. The present decoding mechanism further establishes that discrimination between tRNAs is primarily founded on spatial fit^29^,^30^,^48^ rather than on the number of hydrogen bonds between the ‘closed' decoding centre and the codon–anticodon duplex^35^.

## Methods

### Materials

Uncharged, native individual tRNA^Lys^_SUU_ and tRNA^fMet^_CAU_ from *E. coli* were purchased from Chemical Block (Russia). The mRNA constructs whose sequences are specified below were from Thermo Scientific (USA) and deprotected following the supplier procedure. All mRNA constructs contained identical sequence 5′-GGCAAGGAGGUAAAA-3′ at the 5′-end, which was followed by 5′-AUGAAAA_6_-3′ (mRNA-1), 5′-AUGAAGA_9_-3′ (mRNA-2), 5′-AUGUAAA_9_-3′ (mRNA-3) or 5′-AUGAUAA_9_-3′ (mRNA-4). Aminoglycoside antibiotic paromomycin was purchased from Sigma-Aldrich.

### Purification of the ribosomes

Purification of the 70S ribosomes from strain HB8 of *T. thermophilus* was performed according to the protocol described in ref. 30.

### Complex formation

All ribosomal complexes were formed in 10 mM Tris-acetate pH 7.0, 40 mM KCl, 7.5 mM Mg(CH_3_COO)_2_, 0.5 mM dithiothreitol at 37 °C. For the cognate complexes (Fig. 1c, complexes 1 and 2), the 70S ribosomes (3 μM) were pre-incubated with fivefold excess of mRNA-1 or mRNA-2 and threefold excess of tRNA^fMet^_CAU_ for 15 min to fill the P-site. Then, tRNA^Lys^_SUU_ was added at fivefold excess and incubation was continued for 30 min. Near-cognate complexes (Fig. 1c, complexes 3 and 4) were prepared in a similar manner with the use of mRNA-3 and mRNA-4 constructs. Complexes with paromomycin were obtained by including the antibiotic (60 μM) into the incubation mixture containing 70S/tRNA^fMet^/mRNA-3/tRNA^Lys^_SUU_ or 70S/tRNA^fMet^/mRNA-4/tRNA^Lys^_SUU_.

### Crystallization and crystal treatment

Crystals were grown at 24 °C via vapour diffusion in sitting-drop plates (CrysChem, Hampton Research). The ribosomal complex (2 μl) containing 2.8 mM Deoxy Big Chaps (CalBioChem) was mixed with the equal volume of the crystallization solution (3.9–4.2% (w/v) PEG 20k, 3.9–4.2% (w/v) PEG550mme, 100 mM Tris-acetate pH 7.0, 100 mM KSCN). The crystals grew for 2–3 weeks and were then dehydrated by exchanging the reservoir for 60% (v/v) 2-methyl-2,4-pentanediol. Before freezing in the nitrogen stream, crystals were then cryoprotected by the addition of 30% (v/v) 2-methyl-2,4-pentanediol and 14 mM Mg(CH_3_COO)_2_.

### Structure determination

Data for all complexes were collected at the PXI beamline of Swiss Light Source, Switzerland, at 100 K. A very low-dose mode was used and high redundancy data were collected^52^. The data were processed and scaled using XDS^53^. All crystals belong to space group P2_1_2_1_2_1_ and contain two ribosomes per asymmetric unit. One of the previously published structures^29^, with tRNA, mRNA and metal ions removed, was used for refinement with Phenix^54^. The initial model was placed within each data set by rigid body refinement with each biopolymer chain as a rigid body. This was followed by initial coordinate refinement. The resulting electron density maps were inspected in Coot^55^, and the tRNA and mRNA ligands were built in. During several cycles of manual rebuilding followed by coordinate and isotropic B-factor refinement, magnesium ions were added and the final refinement round took place. The data collection and refinement statistics are presented in Table 1.

## Additional information

**Accession codes:** The atomic coordinates and structure factors for the reported crystal structures have been deposited in the Protein Data Bank under accession codes: 5E7K, 5E81, 5EL4, 5EL5, 5EL6 and 5EL7.

**How to cite this article:** Rozov, A. *et al.* Novel base-pairing interactions at the tRNA wobble position crucial for accurate reading of the genetic code. *Nat. Commun.* 7:10457 doi: 10.1038/ncomms10457 (2016).

## Supplementary Material

Supplementary Information Supplementary Figures 1-4

## Figures and Tables

**Table 1:** Data collection and refinement statistics.

	**Complex 1**^a^	**Complex 2**^b^	**Complex 3**^c^	**Complex 4**^d^	**Complex 3+Paro**^e^	**Complex 4+Paro**^f^
PDB ID	5E7K	5E81	5EL4	5EL5	5EL6	5EL7
						
*Data collection*						
Space group	P2_1_2_1_2_1_	P2_1_2_1_2_1_	P2_1_2_1_2_1_	P2_1_2_1_2_1_	P2_1_2_1_2_1_	P2_1_2_1_2_1_
Cell dimensions						
*a*, *b*, *c* (Å)	209.5, 450.1, 621.6	209.2, 448.5, 619.9	209.5, 448.4, 617.9	208.4, 447.1, 616.9	208.4, 446.7, 618.4	210.1, 449.7, 618.6
*α*, *β*, *γ* (°)	90.0 90.0, 90.0	90.0, 90.0, 90.0	90.0, 90.0, 90.0	90.0, 90.0, 90.0	90.0, 90.0, 90.0	90.0, 90.0, 90.0
Resolution (Å)	200–3.2 (3.3–3.2)*	170–2.95 (3.03–2.95)	200–3.15 (3.25–3.15)	200–3.15 (3.25–3.15)	200–3.1 (3.2–3.1)	175–3.05 (3.15–3.05)
*R*_means_	37.7 (435.2)	32.2 (523.7)	31.6 (397.6)	23.3 (487.2)	29.4 (410.4)	30.4 (513.1)
*I*/σ*I*	15.41 (1.10)	15.38 (1.03)	17.02 (0.97)	12.88 (1.07)	12.42 (0.90)	13.29 (1.00)
CC (1/2)^56^	99.9 (37.2)	99.8 (50.4)	99.6 (30.7)	100 (31.8)	99.9 (24.6)	100 (34.1)
Completeness (%)	100 (100)	100 (100)	100 (100)	100 (100)	99.9 (99.5)	100 (100)
Redundancy	108.7 (40.1)	55.9 (55.9)	109.9 (20.0)	56.5 (33.3)	53.9 (16.9)	75.2 (45.6)
						
*Refinement*						
Resolution (Å)	173.719–3.2	147.011–2.95	151.531–3.15	152.436–3.15	151.238–3.1	152.167–3.05
No. reflections	955,729	1,208,284	987,228	982,230	1,028,648	998,508
*R*_work_/*R*_free_	19.14/25.79	19.58/24.18	19.34/25.11	19.35/25.26	19.59/24.88	19.16/24.75
No. atoms						
RNA	202,855	203,676	202,753	202,446	202,912	202,901
Protein	89,071	89,085	88,352	87,585	89,034	89,065
Ligand/ion/water	2,521	5,145	3,200	2,576	2,311	4,218
B*-factors*						
RNA	105.95	97.02	104.66	130.45	104.43	101.83
Protein	111.70	102.14	110.09	135.14	110.51	105.87
Ligand/ion/water	79.81	73.57	79.73	97.69	80.24	77.76
Root mean squared deviations						
Bond lengths (Å)	0.010	0.012	0.010	0.010	0.009	0.011
Bond angles (°)	1.670	2.054	1.715	1.728	1.619	1.842

Number of crystals used for data collection: ^a^11; ^b^3; ^c^12; ^d^8; ^e^6; ^f^8.

^*^Values in parentheses are for highest-resolution shell.
