# Supplementary material for: Novel base-pairing interactions at the tRNA wobble position crucial for accurate reading of the genetic code
Source: Nat Commun. 2016 Jan 21;7:10457. doi: 10.1038/ncomms10457 (PMC4736104; doi:10.1038/ncomms10457)
Supplement: Supplementary Information — Supplementary Figures 1-4 [file ncomms10457-s1.pdf]

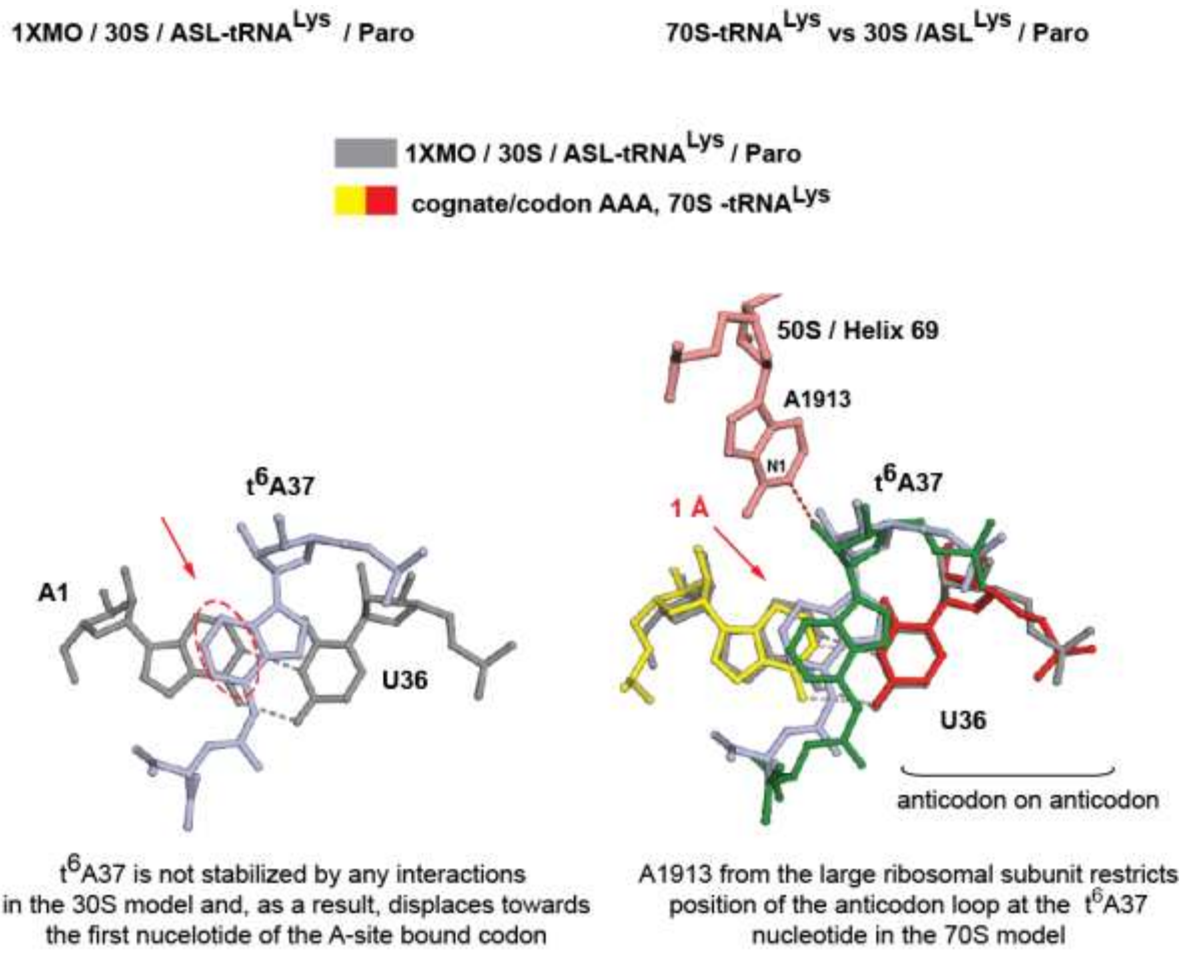

Supplementary Figure 1. Different conformations of *E.coli* tRNA$^{Lys}{}_{UUU}$ anticodon loops in the partial model of the 30S subunit and complete structure of the 70S ribosome. Comparison of the t^{6}A37 stacking interactions in the model of the 30S subunit, which crystals were soaked with the synthetic ASL of *E. coli* tRNA$^{Lys}{}_{UUU}$, hexanucleotide as an analogs of mRNA and miscoding antibiotic paromomycin (Paro)[24], and complete system of the 70S ribosome co-crystallized with native *E. coli* tRNA$^{Lys}{}_{SUU}$ and long mRNA (current study; complex 2). The superposition shows a shift of t^{6}A37 towards the first codon nucleoside in the 30S structure relatively to the position of t^{6}A37 in the 70S model. The observed difference can be explained by the physical lack of a natural constraint from the large ribosomal subunit, i.e. fixation of t^{6}A37 by A1913 from 23S rRNA, on the tRNA^{Lys} ASL in the 30S model.

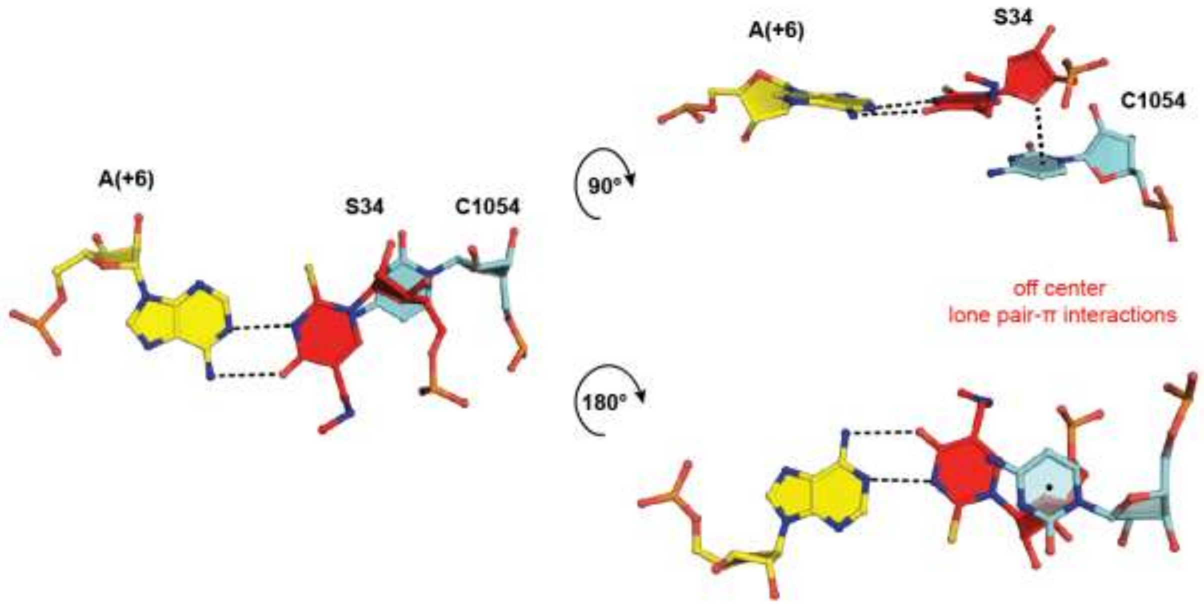

Supplementary Figure 2. Lone pair-aromatic interactions between C1054 and S34. Decoding center on the 70S ribosome controls the first anticodon nucleotide position S34 by weak lone pair-π interactions with ribose of C1054 in 16S rRNA. Displayed are three projections, which show that the specified interaction is off-center.

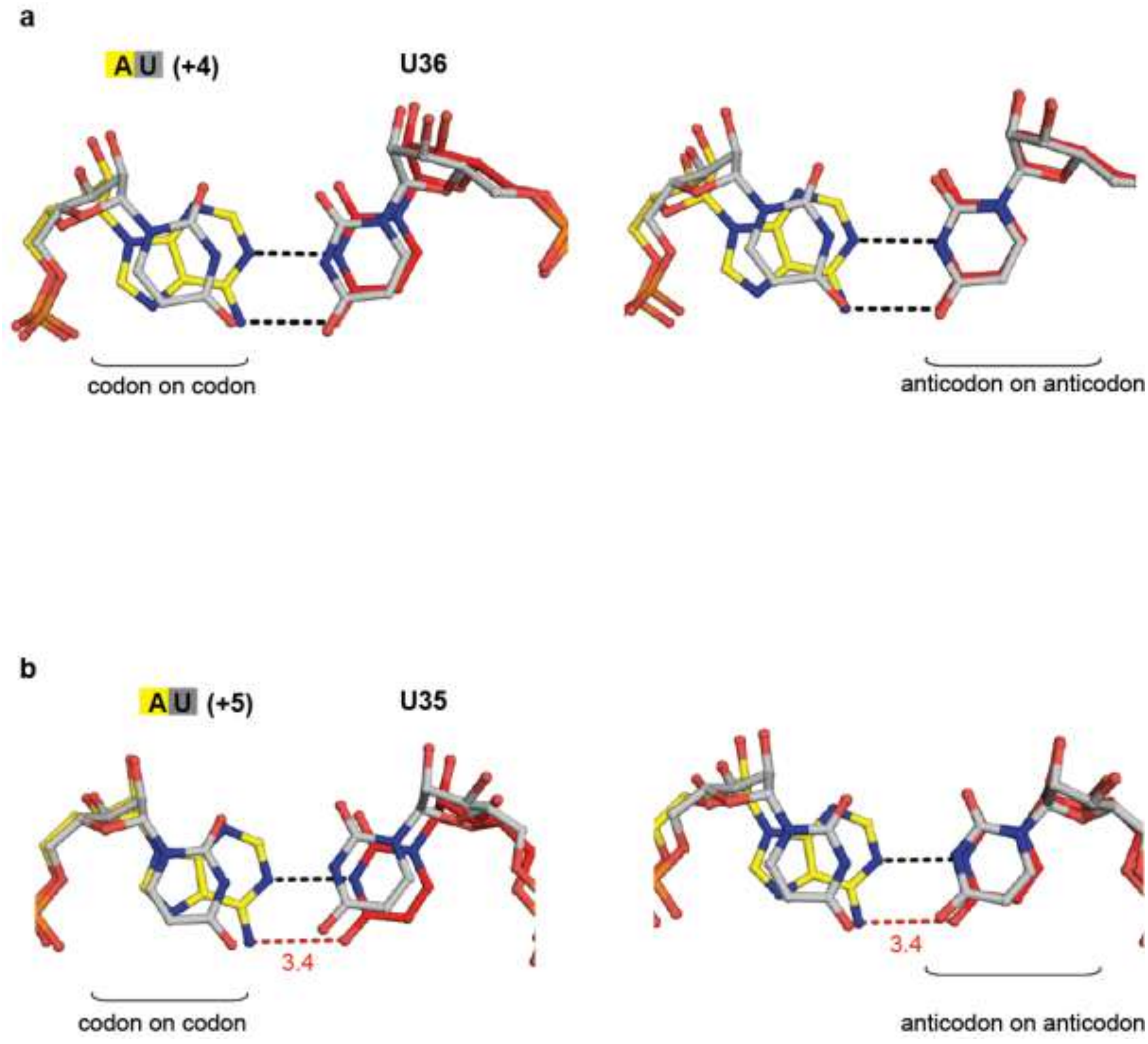

Supplementary Figure 3. Comparison of a pyrimidine-pyrimidine mismatch with the canonical Watson-Crick pair in the decoding center of the 70S ribosome. (**a, b**) Alignment of the Watson-Crick U•A pairs at the first (a, complex 1) and second (b, complex 2) codon-anticodon positions with similar structures where the codon nucleotide was substituted to uridine (complexes 3 and 4). In both U•U mismatches the interatomic distances between Watson-Crick edges of uridines are considerably larger than in canonical pairs (see the main text).

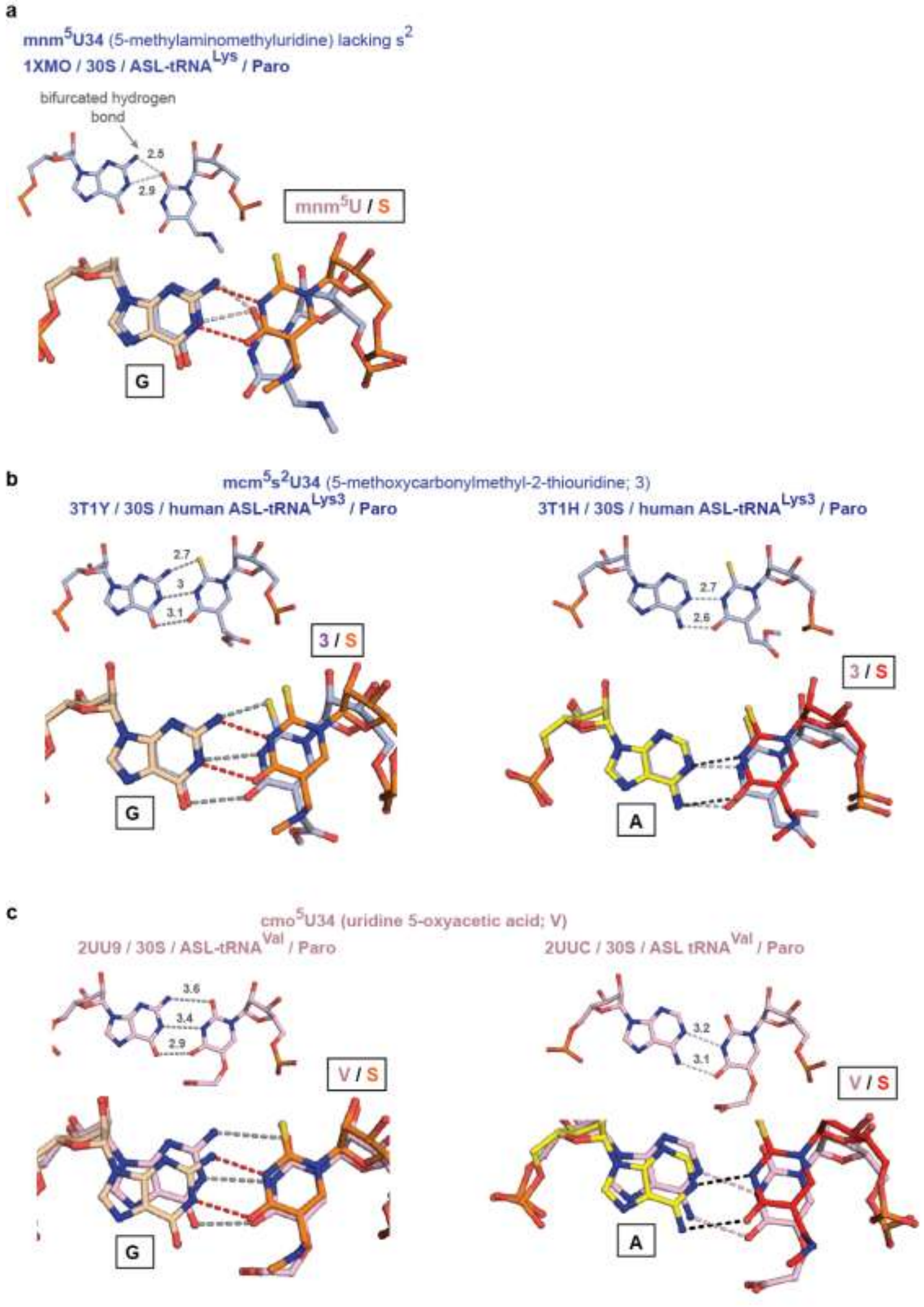

Supplementary Figure 4. Comparison of S34•G and S34•A with other wobble pairs formed by hypermodified uridines. (**a**) Lack of the thio group in mnm^{5}s^{2}U leads to a drastic change in position of the uracil ring in the 30S model; refer to ref. 25, where this structural data are challenged by the same authors. (**b**) The mcm^{5}s^2 modification specific to one of the

eukaryotic species of lysine tRNA induces a Watson-Crick-like conformation of the mcm^5s^2U•G wobble pair in the heterologous system with the bacterial small subunit (left). (**c**) The cmo^5U34 of *E.coli* $tRNA^{Val}_{UAC}$ stimulates a C•G-like canonical interaction in the decoding center of the 30S subunit (left). In (b) and (c) the right panels show alignments of the S34•A pair with mcm^5s^2U34•A and cmo^5U34•A. In all cases the comparison is done by superposition of codons; each panel specifies the PDB code taken for comparison as well as the modification in ASL of tRNA; Paro stands for a miscoding antibiotic paromomycin used in all models of the 30S subunit to improve ASL binding. The difference in atom positions are indicated by arrows and corresponding value in angstroms.
